# Impact of Preoperative Nutritional Status on Postoperative Outcomes in Elective Brain Tumor Surgery: A Retrospective Cohort Study in the Philippines

**DOI:** 10.7759/cureus.94301

**Published:** 2025-10-10

**Authors:** Mamerto Marvin N Buban, Jaime E Rama

**Affiliations:** 1 Neurosurgery, Vicente Sotto Memorial Medical Center, Cebu City, PHL

**Keywords:** brain tumor, elective craniotomy, malnutrition, neurosurgery outcomes, nutritional status, philippines

## Abstract

Introduction: Malnutrition, encompassing undernutrition and overnutrition, is a modifiable risk factor for adverse surgical outcomes. While extensive literature links nutritional deficits to poor perioperative outcomes in general surgery, the role of nutrition in elective neurosurgery remains insufficiently characterized, particularly in low- and middle-income countries. This study examined the relationship between preoperative nutritional status and postoperative outcomes in patients undergoing elective brain tumor surgery in the Philippines. Methods: A retrospective cohort study was conducted among adult patients (18-60 years) who underwent elective craniotomy for brain tumor excision at Vicente Sotto Memorial Medical Center (VSMMC) between December 2021 and December 2022. Patients were stratified by body mass index (BMI) into undernourished (<18.5 kg/m^2^), well-nourished (18.5-24.9 kg/m^2^), and overnourished (>25 kg/m^2^). Outcomes included in-hospital mortality, length of hospital stay, and postoperative infections. Statistical analyses employed t-tests, Mann-Whitney U tests, chi-square tests, and Fisher’s exact tests, with significance set at p<0.05. Results: A total of 94 patients were included: six undernourished, 44 well-nourished, and 44 overnourished. Undernourished patients experienced significantly higher mortality compared to well-nourished patients (p=0.0176), while overnourished patients exhibited the highest absolute mortality (7%). No significant differences were observed in infection rates or hospital stay across groups. Conclusion: Preoperative malnutrition is associated with increased in-hospital mortality in elective brain tumor surgery. Routine nutritional assessment and optimization should be incorporated into preoperative planning to improve neurosurgical outcomes.

## Introduction

Malnutrition, broadly defined as an imbalance in nutrient intake, includes both undernutrition and overnutrition. Globally, an estimated 462 million adults are underweight, while 1.9 billion are overweight or obese [1]. In the Philippines, chronic undernutrition has persisted for decades even as obesity and diet-related non-communicable diseases rise, creating a dual burden of malnutrition [2]. The adverse effects of malnutrition on surgical outcomes are well-documented. Malnourished patients experience higher complication rates, more extended hospital stays, and increased mortality [3,4]. Economic analyses further highlight the burden, with hospital malnutrition associated with increased costs and prolonged hospital admissions [5-7]. In cancer surgery, malnutrition worsens wound healing and survival, prompting calls for routine perioperative nutrition support [8,9]. In neurosurgery, patients are uniquely vulnerable due to the high metabolic demands of central nervous system pathology and recovery. Malnutrition has been shown to impair immune function, reduce physiological reserve, and increase the risk of infection [10-13]. A study demonstrated poorer recovery in malnourished patients, particularly in emergency settings [14]. Yet, the impact of nutritional status in elective brain tumor resections remains underexplored. Given the persistent burden of malnutrition in low- and middle-income countries and the scarcity of data in elective neurosurgery, this study aimed to investigate the relationship between preoperative nutritional status and postoperative outcomes in patients undergoing elective brain tumor surgery at a Philippine tertiary hospital.

Objectives

General Objective

The general objective was to evaluate the impact of preoperative nutritional status on postoperative outcomes in patients undergoing elective brain tumor surgery.

Specific Objectives

The specific objectives included: (i) characterizing the demographic and clinical profiles of undernourished, well-nourished, and overnourished patients; and (ii) comparing postoperative outcomes - including mortality, infection rates, and length of hospital stay - across defined nutritional status groups, as well as evaluating the statistical significance of observed differences in these outcomes.

## Materials and methods

Study design and setting

A retrospective cohort study was conducted at Vicente Sotto Memorial Medical Center (VSMMC), a 1,500-bed tertiary government hospital in Cebu City, Philippines.

Population

Inclusion Criteria

All patients aged 18-60 years who underwent elective craniotomy for brain tumor excision between December 2021 and December 2022 were included in the study.

Exclusion Criteria

Patients with ongoing/active infection requiring antibiotic treatments, those who underwent cranial reoperations during the same confinement, or individuals with known nutritional disorders (e.g., rickets, scurvy, iron deficiency anemia, goiter) were excluded from the study.

Variables

The nutritional status of the patients was classified by body mass index (BMI) (15 kg/m^2^): undernourished (<18.5 kg/m^2^), well-nourished (18.5-24.9 kg/m^2^), and overnourished (>25 kg/m^2^). The study outcomes included in-hospital mortality, length of stay, surgical site infection, hospital-acquired pneumonia, ventilator-associated pneumonia, and catheter-associated urinary tract infection (UTI).

Statistical analyses

Descriptive statistics were utilized to summarize the demographic and clinical profiles of the participants. The Shapiro-Wilk test was used to assess normality. Independent t-tests or Mann-Whitney U tests were applied for continuous variables, while the chi-square tests or Fisher’s exact tests were employed for categorical variables. Statistical significance was set at p<0.05. All analyses were performed using RStudio v4.2.0 (RStudio, Inc., Boston, MA, USA).

Ethical considerations

The VSMMC Research Institute (VRI) and Research Ethics Committee (VSMMC-REC) approved the study. Patient confidentiality was ensured by anonymization.

## Results

Table 1 shows the clinical profile of 94 patients: six (6%) were undernourished, 44 (47%) were well-nourished, and 44 (47%) were overnourished. Female patients made up 69% of the group. Hypertension (35%) and diabetes mellitus (21%) were common, particularly among overnourished patients. Undernourished patients had lower serum albumin levels compared to the other groups.

**Table 1:** Demographic and clinical profile of patients (N=94)

Variable	Overall (n=94)	Undernourished (n=6)	Well-nourished (n=44)	Overnourished (n=44)	P-value
Age (years), mean ± SD	43.20 ± 12.15	41.67 ± 13.15	42.52 ± 12.60	44.05 ± 11.94	0.83
Weight (kg), mean ± SD	57.02 ± 12.88	40.33 ± 6.53	52.43 ± 8.33	64.41 ± 11.90	<0.0001
Albumin (g/dL), mean ± SD	3.00 ± 0.52	2.80 ± 0.40	3.00 ± 0.26	3.00 ± 0.66	0.48
Sex: male, n (%)	29 (31%)	1 (17%)	12 (27%)	16 (36%)	0.48
Sex: female, n (%)	65 (69%)	5 (83%)	32 (73%)	28 (64%)	–
Hypertension, n (%)	33 (35%)	2 (33%)	13 (30%)	18 (41%)	0.47
Diabetes mellitus, n (%)	20 (21%)	1 (17%)	6 (14%)	13 (30%)	0.16

Table 2 reveals that hospital stays were longest among undernourished patients (14.17 ± 8.61 days) and shortest in well-nourished patients (10.77 ± 8.07 days), though the difference was not statistically significant. Overall, 20% of patients experienced at least one infectious complication, with hospital-acquired pneumonia being the most common (9%). In-hospital mortality occurred in 13 patients (≈14%). Undernourished patients demonstrated significantly greater mortality compared with well-nourished patients (p=0.0176). Overnourished patients had the highest absolute mortality rate (7%).

**Table 2:** Association between nutritional status and clinical outcomes *Significant difference between undernourished and well-nourished groups (p<0.05, Fisher’s exact test). UTI: Urinary tract infection

Outcome	Undernourished (n=6)	Well-nourished (n=44)	Overnourished (n=44)	P-value
Length of stay (days), mean ± SD	14.17 ± 8.61	10.77 ± 8.07	12.39 ± 8.22	0.53
In-hospital mortality, n (%)	3 (50%)	3 (7%)	7 (16%)	0.0176*
Surgical site infection, n (%)	0	2 (5%)	1 (2%)	0.72
Hospital-acquired pneumonia, n (%)	1 (17%)	3 (7%)	5 (11%)	0.65
Ventilator-associated pneumonia, n (%)	0	2 (5%)	2 (5%)	0.84
Catheter-associated UTI, n (%)	0	2 (5%)	1 (2%)	0.72

## Discussion

This study found that preoperative malnutrition significantly increased in-hospital mortality in patients undergoing elective brain tumor surgery, even when infection rates and hospital stays were not significantly affected. These findings underscore nutritional status as an underrecognized but critical determinant of neurosurgical outcomes. The results align with global evidence that malnutrition worsens surgical outcomes [3,4,15]. Research has demonstrated its independent impact on morbidity and mortality [3], while another study highlighted increased costs and hospital stays in malnourished patients [15]. In cancer surgery, it has been confirmed that malnutrition delays recovery and compromises survival [8,9]. Our findings extend this evidence into elective neurosurgery, a field with limited dedicated studies. Neurosurgical-specific literature emphasizes the dual burden of malnutrition. Studies have identified both underweight and obesity as risk factors for infection and poor healing [5,6], and poorer outcomes in emergency neurosurgical cases have been reported [14]. By focusing on elective craniotomies, this study reveals that malnutrition confers mortality risk even in preoperatively optimized patients. The higher absolute mortality in overnourished patients (7%) echoes the "obesity paradox" described in surgical populations. A study found that obesity increases the risk of complications after spine surgery [12]. In our cohort, obesity correlated with higher rates of hypertension and diabetes, likely amplifying perioperative risk. These results support the view that both undernutrition and overnutrition act as independent risk modifiers in neurosurgery. Infection rates and hospital stays did not differ significantly across groups, diverging from prior findings [9,15]. Possible explanations include the small sample size of undernourished patients, uniform infection control protocols at VSMMC, and population-specific differences. In the Philippines, where chronic undernutrition coexists with rising obesity, BMI alone may inadequately reflect metabolic reserve. Alternative measures such as subjective global assessment [16,17], serum albumin, and anthropometric tools may provide a more nuanced assessment. From a clinical perspective, nutritional screening should be integrated into preoperative neurosurgical protocols. The European Society for Clinical Nutrition and Metabolism (ESPEN) guidelines recommend routine nutritional evaluation in surgical patients [18]; however, this remains underutilized in practice, especially in low- and middle-income countries. Economic studies [5-7] further argue for its cost-effectiveness. Future research should investigate prospective interventions, including immunonutrition and prehabilitation, to determine whether nutritional optimization improves neurosurgical survival.

Limitations

This study has several limitations. First, the undernourished group comprised only six patients, limiting the statistical power and generalizability of the subgroup findings. Second, the retrospective design may have introduced selection and documentation biases. Third, nutritional status was assessed using BMI alone, which does not account for sarcopenia, micronutrient deficiencies, or functional status; tools such as subjective global assessment may provide greater sensitivity. Additionally, the absence of adjustment for potential confounders such as tumor type, grade, and perioperative clinical factors, which may have influenced outcomes independently of nutritional status, was not considered. Finally, this was a single-center study in a Philippine tertiary hospital, and the results may not fully represent other neurosurgical populations. Future multicenter, prospective studies with larger cohorts and comprehensive nutritional assessments are needed to validate these findings.

## Conclusions

Preoperative malnutrition is associated with increased in-hospital mortality in elective brain tumor surgery. Both undernutrition and overnutrition confer risk, emphasizing the importance of routine nutritional screening and targeted interventions in preoperative neurosurgical care.

## References

[1] (2025). Malnutrition. https://www.who.int/news-room/fact-sheets/detail/malnutrition.

[2] Adona AJV, Demombynes G, Mbuya NV, Piza SFA (2021). Undernutrition in the Philippines: Scale, Scope, and Opportunities for Nutrition Policy and Programming (English). http://documents.worldbank.org/curated/en/798061619807652635.

[3] Correia MI, Waitzberg DL (2003). The impact of malnutrition on morbidity, mortality, length of hospital stay and costs evaluated through a multivariate model analysis. Clin Nutr.

[4] Norman K, Pichard C, Lochs H, Pirlich M (2008). Prognostic impact of disease-related malnutrition. Clin Nutr.

[5] Curtis LJ, Bernier P, Jeejeebhoy K (2017). Costs of hospital malnutrition. Clin Nutr.

[6] Amaral TF, Matos LC, Tavares MM, Subtil A, Martins R, Nazaré M, Sousa Pereira N (2007). The economic impact of disease-related malnutrition at hospital admission. Clin Nutr.

[7] Álvarez-Hernández J, Planas Vila M, León-Sanz M (2012). Prevalence and costs of malnutrition in hospitalized patients; the PREDyCES® study. Nutr Hosp.

[8] Previtali P, Fiore M, Colombo J (2020). Malnutrition and perioperative nutritional support in retroperitoneal sarcoma patients: results from a prospective study. Ann Surg Oncol.

[9] Sandrucci S, Cotogni P, De Zolt Ponte B (2020). Impact of artificial nutrition on postoperative complications. Healthcare (Basel).

[10] Dobner J, Kaser S (2018). Body mass index and the risk of infection - from underweight to obesity. Clin Microbiol Infect.

[11] Tahta A, Turgut YB, Sahin C (2021). Malnutrition essentials for neurologists and neurosurgeons: a review of the literature. J Pediatr Neurol.

[12] Johnson KG, Alsoof D, McDonald CL, Berreta RS, Cohen EM, Daniels AH (2022). Malnutrition, body mass index, and associated risk of complications after posterior lumbar spine fusion: a 3:1 matched cohort analysis. World Neurosurg.

[13] Dionyssiotis Y, Papachristos A, Petropoulou K, Papathanasiou J, Papagelopoulos P (2016). Nutritional alterations associated with neurological and neurosurgical diseases. Open Neurol J.

[14] Freitas MM, Stanich P, Diccini S (2019). Status and nutritional therapy in elective and emergency neurosurgery patients. Rev Bras Enferm.

[15] Subwongcharoen S, Areesawangvong P, Chompoosaeng T (2019). Impact of nutritional status on surgical patients. Clin Nutr ESPEN.

[16] Detsky AS, McLaughlin JR, Baker JP, Johnston N, Whittaker S, Mendelson RA, Jeejeebhoy KN (1987). What is subjective global assessment of nutritional status?. JPEN J Parenter Enteral Nutr.

[17] da Silva Fink J, Daniel de Mello P, Daniel de Mello E (2015). Subjective global assessment of nutritional status - a systematic review of the literature. Clin Nutr.

[18] Kondrup J, Allison SP, Elia M, Vellas B, Plauth M (2003). ESPEN guidelines for nutrition screening 2002. Clin Nutr.

